# How is range of motion of the fingers measured in hand therapy practice? A survey study

**DOI:** 10.1177/17589983241237780

**Published:** 2024-04-22

**Authors:** Zeal Kadakia, Sandra VanderKaay, Ayse Kuspinar, Tara Packham

**Affiliations:** School of Rehabilitation Science, Institute for Applied Health Sciences, McMaster University, Hamilton, ON, Canada.

**Keywords:** Range of motion, hand therapy, finger, survey

## Abstract

**Introduction:**

A variety of techniques for measuring finger range of motion (ROM) are available for hand therapist use, however, there is no clear description of which finger ROM methods are preferred in practice. This study explored the preferred measurement techniques, the factors influencing clinical decision-making, and the clinical reasoning processes employed when faced with practice-based measurement scenarios.

**Methods:**

This was a cross-sectional online survey study of hand therapists and American or Canadian Society of Hand Therapists members. Quantitative methods were employed for participant demographics and categorical clinical questions about practice patterns. Qualitative descriptive questions and vignettes were analysed using inductive and deductive content analysis, respectively.

**Results:**

Four hundred and eighty-one responses were included, representing hand therapists with a median age of 51 years and median experience of 19 years. Participants preferred measuring individual joints with a goniometer (*N* = 210, 44%) for perceived utility in informing treatment decisions, reliability, and confidence in measurement skills. Participants also preferred active functional ROM (*N* = 117, 24%) for being quick, easy, and useful in informing treatment decisions. Participants reported using different methods with time constraints in a busy clinic, taking precautions with pins/wounds, bulky dressings/casts, pain tolerance levels of patients, or with specific pathologies. Participants’ responses to the multi-stage vignette identified distinct patterns of clinical reasoning approaches within individual vignettes.

**Conclusions:**

Hand therapists use multiple methods to measure finger ROM while preferring to use goniometers to measure individual finger joints. They engage procedural and pragmatic reasoning modified by contextual factors when measuring finger ROM.

## Introduction

Measuring active and passive range of motion (ROM) with a goniometer is a foundational skill for occupational therapists and physiotherapists.^1,2^ In hand therapy, these measures are used to establish baseline status, inform treatment selection, evaluate treatment response, inform orthosis design, and monitor progress.^3^

Recommendations from the American Society of Hand Therapists^4^ describe a broad spectrum of techniques for measuring finger motion but it is unclear (a) what methods are used in hand therapy settings, and (b) if the published literature reflects standardised procedures for research or the daily practices of busy therapists. For example, a measurement review of tendon and nerve study outcomes^5^ reported common measures were individual joint ROM, summary joint motion (total active motion: TAM), palm-to-pulp distance, and categorical ratings (e.g. Strickland). A recent practice survey of certified hand therapists^6^ asked broad questions about upper extremity evaluation and did not specify the tools or methods used for finger ROM measurement. There has not been a clear description of the potential factors influencing the choice of measurements for finger ROM, including practice setting, clinician experience, client diagnoses, treatment objectives, clinical reasoning, equipment availability, policies and documentation requirements, and the inherent time and skill demands of the measurement techniques themselves.

Higgs and Jones^7^ define clinical reasoning (or practice decision making) as “a context-dependent way of thinking and decision making in professional practice to guide practice actions… within a set of problem spaces informed by the practitioner’s unique frames of reference, workplace context and practice models, as well as by the patient’s or client’s contexts”^7^. Vignettes are a valuable tool in research for questions investigating accounts of practice and influencing factors^8^; they are brief, hypothetical scenarios providing context and information to explore clinical reasoning. We used vignettes to explore the clinical reasoning of hand therapists, drawing on an adapted Four-Part Framework for Clinical Reasoning ^9,10^ where *procedural reasoning* involves a focus on the disease, limitations, or disabilities of the client. This involves a dual search for problem definition and treatment selections: therapists make quick decisions based on information related to a diagnosis. *Pragmatic reasoning* involves considerations beyond the client-therapist interaction, including the client’s context, the therapist’s knowledge, values and abilities, and organizational barriers and facilitators. *Interactive reasoning* focuses on the client as a person, considering their point of view and illness experience, including psychosocial needs and goals. In *conditional reasoning*, the therapist considers the client’s current and future context and demands of needed or desired occupations; how the client’s condition may change over time; and how future contexts may support or limit occupational engagement.^9,10^

Specifically, we explored what measures are used in clinical practice, and the factors contributing to these practice patterns. This information can help inform the prioritisation of measurement studies to address gaps in the psychometric literature and identify opportunities for knowledge translation and continuing education.

We used data gathered from a survey including standardised demographic and clinical practice setting questions,^11^ specific practice questions and vignettes intended to elicit clinical reasoning and contextual factors.^8^

Our primary objective was to explore the methods used by hand therapists to measure ROM of the fingers in practice. Our secondary objectives were to elucidate clinical reasoning including contextual factors and the influence of practice characteristics by answering the following questions:

(1) What factors influence therapists’ clinical decision-making around finger ROM measurement?
(2) How do therapists present their clinical reasoning when faced with finger ROM measurement scenarios?

## Methods

### Study design

This was a cross-sectional study, using an online survey with both discrete choice and open-text responses examining ROM measurement practices and clinical reasoning around evaluation. Data were analysed using a mixed methods approach^12^ to answer our research questions, embedding qualitative components within a quantitative design. We have reported this study guided by Checklist for Reporting Results of Internet E-Surveys^13^ and Consensus-Based Checklist for Reporting of Survey Studies^14^; see appendix A.

### Data collection methods

The survey had three sections on three screens/pages^11^: (1) 16 sociodemographic questions addressing personal and professional characteristics, (2) three clinical vignettes on measurement choices for specific cases and (3) thirty-one fixed choice and open-ended survey questions regarding upper extremity ROM measurement preferences and practices. Fixed choice questions around finger ROM measurement practices included: Individual, isolated ranges for metacarpophalangeal, proximal interphalangeal, distal interphalangeal (MCP, PIP, DIP) joints with a goniometer, Strickland ratings, Total active motion (TAM) = a summary of active flexion and extension, Total passive motion (TPM) = a summary of passive flexion and extension, Functional range of motion (FROM)/pulp-to-palm/composite finger flexion (CFF) = active combined flexion measured with ruler as distance between fingertip and distal palmar crease (in cm), FROM/CFF passive = combined passive flexion measured with ruler as distance between fingertip and distal palmar crease (in cm), wire bending (solder wire shaped to finger), paper strip method, and ‘Eyeball’ or visual estimate. To address our secondary objective of understanding factors influencing clinical decision-making around finger ROM measurement, we developed a set of sub-questions to guide our analysis:

(a) Why do they utilise a particular method?
(b) When and why do they use other methods?
(c) What are these ROM measurements used for in their practice?
(d) Has their practice changed over time?
(e) Are therapist assistants included in ROM measurement in their setting?
(f) Are clients asked to employ self-monitoring techniques?

This survey was developed and piloted using multiple techniques, including expert review and cognitive debriefing, and underwent vetting by the American Society of Hand Therapists (ASHT) before distribution (copy available in supplemental materials).^11^ While the full survey explored measurement choices for fingers, hand, and wrist, we will only report the data regarding finger ROM here, with the intent of sharing the remaining data in a separate report.

### Sample characteristics

The sample for this study was purposive^15^: we included occupational therapists, physiotherapists/physical therapists, and any health professionals currently treating clients with hand and/or wrist injuries in a hand therapy role in any setting. Respondents were excluded if they were retired, were in a solely academic role, or were not currently treating persons with hand injuries as a core population. Responses were not excluded based on geographical location outside of the US/Canada. We posted the survey link on social media and surveyed members of ASHT and the Canadian Society of Hand Therapists (CSHT) using membership lists where therapists had agreed to receive surveys. At the time of the survey, this represented approximately 200 members of CSHT, and 3000 members of ASHT. Given the low survey response levels (5-30%) documented in other survey studies of this population,^16–18^ we estimated obtaining a sample size of 400 complete responses. Thus, our target response rate was 12.5%.

### Survey administration

Approval for conducting the survey was obtained from the Hamilton Integrated Research Ethics Board (under project #7465), with reciprocal review by New York University, and the research committees of ASHT and CSHT. The survey was administered through LimeSurvey (LimeSurvey GmbH, Hamburg, Germany), hosted on a secure server at McMaster University. The survey was open from March 31, 2020, until August 2, 2020, unfortunately coinciding with the onset of the COVID-19 pandemic. Consequently, we decided to remove the mandated entry of responses for every question on the survey.

### Statistical analysis

Data analysis was conducted on Stata version 18 software (StataCorp, Texas, USA). Descriptive statistics were calculated for participant characteristics and practice settings. To address missing data, item non-response was identified. Survey responses missing data for more than half of the survey were excluded using a complete case analysis (listwise deletion) approach.^19^ Categorical clinical content questions were analysed using descriptive statistics (frequencies) and visualized using bar charts where appropriate. To enhance interpretability, we condensed the original response categories for the survey question *When measuring motion in the hand and fingers, how often have you used the following measurement techniques in the past month?* The original categories, *always use* and *use frequently* were condensed together to form the category Frequently used*, use occasionally* remained as Occasionally used*, use rarely* and *never use*, were collapsed into Rarely or never used. Similarly, for the question *How often do you treat these conditions as the primary diagnosis across your practice settings?,* the original categories *Never (0%)* remained as is, *Rarely (1-25%)* and *Occasionally (26-50%)* were condensed to Occasionally (1-50%), and *Often (51-75%)* and *Routinely (76-100%)* were condensed into Routinely (51-100%).

### Qualitative analysis

Qualitative analysis was conducted by the first author, who holds an “outsider”^20^ perspective with regards to hand therapy, as an internationally educated physiotherapist with general experience. The underlying paradigm for the qualitative analysis was constructivist, acknowledging the presence of multiple realities and emphasizing the co-creation of knowledge through an exploration of participants’ subjective experiences and perspectives. Open text survey responses were read by the first and last authors several times to immerse themselves in the data.^21^ Open text survey responses or responses that were deemed to not answer the question were excluded as missing data after dual review and consensus.

Inductive content analysis^21^ was employed for open-ended qualitative description questions. This technique is useful when there is not enough prior knowledge about the phenomenon.^21^ In this study, we explored participants’ rationale for any changes in measurement method preferences over time or in a given circumstance. After open codes were generated, the coded data were grouped into categories. The categories were then discussed at a team meeting with the last author who possesses an “insider” view of the data (survey designer) and hand therapy. At this stage, the researchers decided to map the codes and categories onto existing theoretical frameworks, namely the COM-B Model for Behaviour Change^22^ and Clinical Decision Making (CDM) in Multiple Problem Spaces,^7^ to engage theoretical triangulation^23^ and support qualitative rigour.^24^

The COM-B model for Behaviour Change^22^ states that *behaviour* is influenced by the interaction of *capability (C), opportunity (O)*, and *motivation (M)*. Within these three elements shaping behaviour, there are six components: physical capability (physical skill), psychological capability (knowledge), physical opportunity (environment), social opportunity (cultural milieu), reflective processes (evaluation and planning), and automatic processes (reflexive impulses).^22^ Key themes generated from the inductive analysis of the participants’ changing measurement preferences over time were mapped onto the six components to describe how their behaviour was influenced.

The Clinical Decision Making (CDM) in Multiple Problem Spaces model^7^ identifies ‘problem spaces’ encompassing various contexts of clinical reasoning. These include the immediate clinical problem, the task environment, practitioner frames of reference, and patient perspectives; and the influence of local, organizational, socio-cultural, and global factors.^7^ This model was used to map codes and categories illustrating contextual factors (or problem spaces) in clinical decision-making (CDM) regarding when participants described choosing to use an alternate method to measure finger ROM.

Deductive content analysis^21^ was used to analyse the multi-stage vignette drawing on the adapted Four-Part Framework for Clinical Reasoning.^9,10^ This framework provides an understanding of the types of clinical reasoning used in hand therapy practice (see supplemental material Figure (A)). An unconstrained categorization matrix was developed in Microsoft Word and data were coded and categorized into the matrix.^21^ Respondents had to answer at least one of three vignette segments for the data to be included in the analysis. Responses to each of the three vignette segments were analysed per segment; then vignette responses from each respondent were examined as a collective response to all three vignettes, followed by a discussion with the senior author to review the analysis and support trustworthiness.^24^

## Results

The survey had 547 respondents, out of which 66 responses were excluded for not meeting the eligibility criteria (missing responses = 40, not currently treating persons with hand injuries = 19, academic role = 7) for a total sample size of *n* = 481. See Table 1 for a complete account of participant demographics.

**Table 1.** Participant characteristics and practice settings (*N* = 481).

Variables	Frequency
Median age (range)	51 (24 – 72)
Sex *N* (%)	
Male	49 (10)
Female	428 (89)
Prefer not to say	4 (0.9)
Profession *N* (%)	
OT	424 (88)
PT	50 (10)
AT	4 (0.8)
Kinesiologist	1 (0.2)
Dual OT/PT	2 (0.4)
Highest level of education *N* (%)	
Bachelor’s degree	197 (41)
Clinical master’s degree (e.g. MScPT, MScOT)	108 (22)
Academic master’s degree (e.g. MSc, MEd. MBA)	96 (20)
Clinical doctoral degree (e.g. DPT, OTD)	51 (11)
College diploma	10 (2)
Academic doctoral degree (e.g. PhD)	5 (1)
Other (postgraduate certification/diploma, multiple bachelor’s degrees)	14 (3)
Median years as a licensed healthcare professional (range)	25 (1-50)
Median years as a hand therapist (range)	19 (1-46)
Status of hand therapy certification with hand therapy Certification Commission *N* (%)	
Current certification	380 (58)
In pursuit of certification	52 (11)
No certification	38 (8)
Certification not endorsed as a qualification in participant’s jurisdiction	5 (1)
Certification not held currently but was held previously	3 (0.6)
Certified through another organization outside North America	3 (0.6)
Country of practice *N* (%)	
United States of America	405 (84)
Canada	56 (11.6)
South Africa	7 (1.5)
United Kingdom	5 (1)
Ireland	2 (0.4)
Singapore	2 (0.4)
Chile	1 (0.2)
Italy	1 (0.2)
Switzerland	1 (0.2)
Argentina	1 (0.2)
Current primary practice setting *N* (%)	
Hospital based outpatient	214 (44.5)
Physician owned outpatient	88 (18.3)
Therapist-owned outpatient	75 (15.6)
Corporate owned freestanding clinic	57 (11.9)
Private practice, independent contractor	19 (4)
Academic-based hospital setting, outpatient clinic	16 (3.3)
Military health system	1 (0.2)
Industry	1 (0.2)
Other (e.g. senior’s home, community-based rehabilitation)	10 (2.1)
Hours/week providing direct care to patients with upper extremity conditions *N* (%)	
0-9 h	36 (7.5)
10-19 h	51 (10.6)
20-29 h	110 (22.9)
30-40 h	244 (50.7)
More than 40 h	40 (8.3)

Key: *N* = number, SD = Standard Deviation, OT = Occupational therapist, PT = Physiotherapist, AT = Athletic Therapist, MScPT = Master of Science in Physical Therapy, MScOT = Master of Science in Occupational Therapy, MSc = Master of Science, MEd = Master of Education, MBA = Master of Business Administration, DPT = Doctor of Physical Therapy, OTD = Doctor of Occupational Therapy, PhD = Doctor of Philosophy.

At least one of three vignette questions was answered by 308 participants while 173 participants did not answer any vignette. Participants who answered vignettes had a mean of 19.8 years of experience (SD = 10.8) while those not answering vignettes had a mean of 17.2 years of experience (SD = 11.1). See supplemental material Figure (B) for a flow chart of participant.

The top five primary diagnoses seen routinely across practice settings were fractures (*n* = 396), cumulative trauma disorders (*n* = 230), arthritis and rheumatic diseases (*n* = 227), ligamentous injuries (*n* = 227), and nerve injuries (*n* = 219). See supplemental material Graph A for a detailed account of the diagnoses and their frequency seen across practice settings.

### Methods and equipment used by hand therapists to measure ROM of the finger

**Figure 1.**
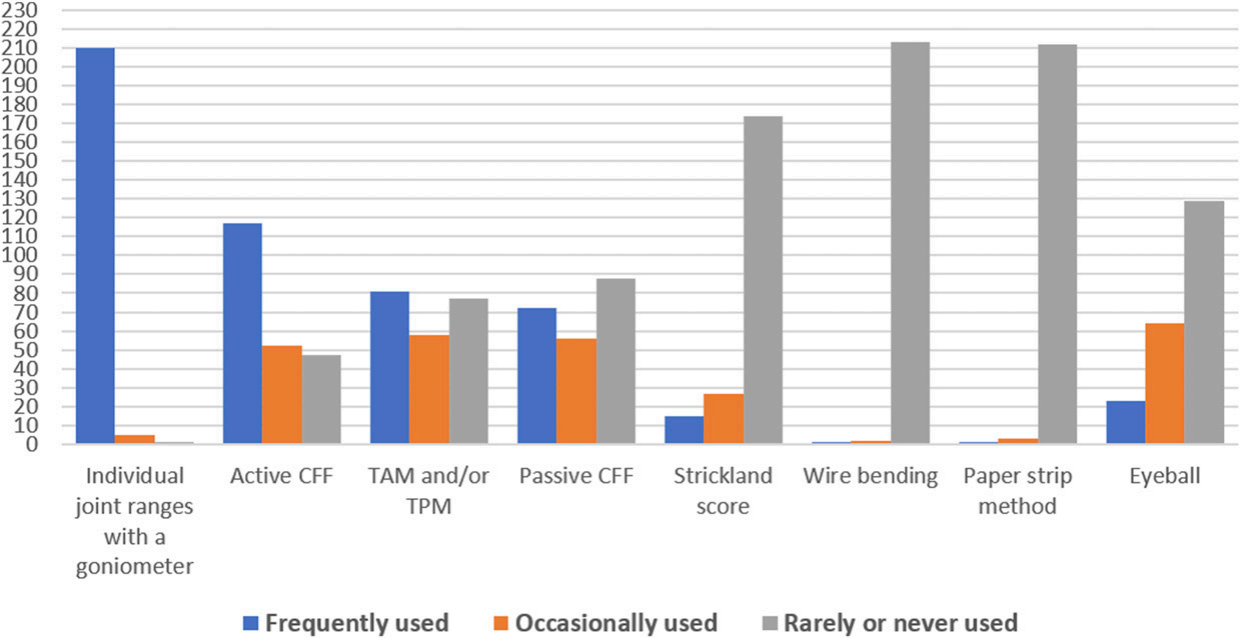
Frequency of use of various finger ROM measurement techniques.

The three most frequently used techniques to measure finger ROM were individual joint ranges with a goniometer (*n* = 210), active functional ROM (*n* = 117), and total active and passive motion (*n* = 81): see Figure 1 for an accounting of all methods and frequency of use.

The most preferred method for measuring finger ROM was individual joint ranges with a goniometer (*n* = 165, 76.4%), followed by active FROM (AFROM) (*n* = 25, 12%), TAM/TPM (*n* = 12, 5%), passive FROM (PFROM) (*n* = 2, 1%) and eyeball (*n* = 2, 1%). Some participants preferred to use other measurement methods (*n* = 10, 5%) not included in the survey such as biometric systems. See supplemental material Graph B for a detailed account of measurement preferences for finger ROM. Finger goniometers, placed on the dorsum of the finger joints with flat arms,^25^ were the most used equipment (*n* = 187, 92.1%) for measuring movement in the hand and fingers, followed by a standard goniometer, or universal goniometer, (*n* = 14, 6.9%): no participants reported using smartphone measurement apps.

### Factors influencing clinical decision-making

**Figure 2.**
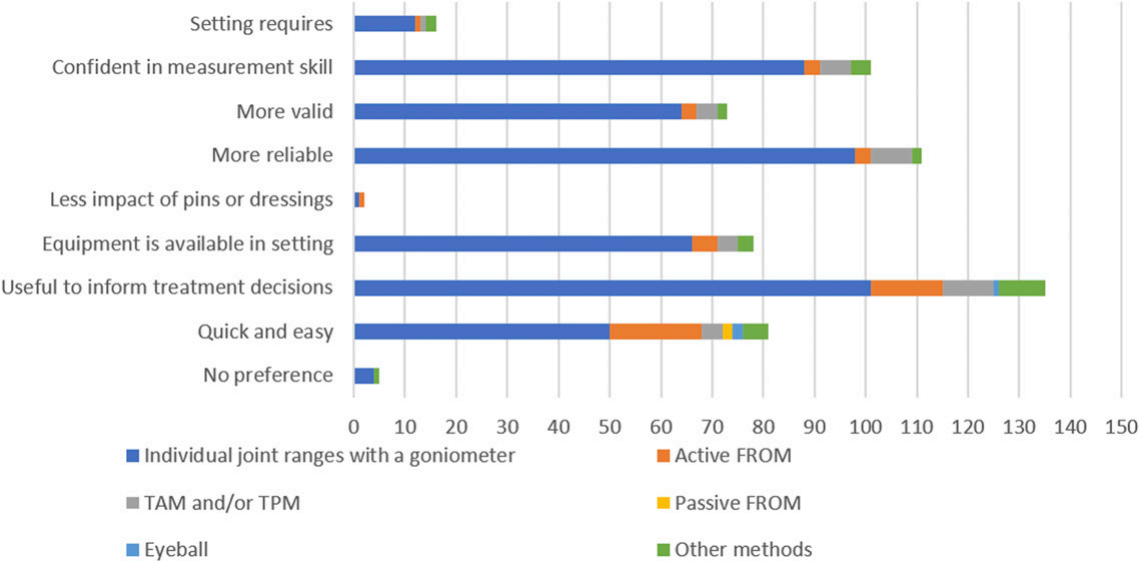
Frequency of reason for choosing a ROM measurement method.

Participants preferred using goniometry to measure individual joint ROM due to its usefulness in informing treatment decisions, reliability, and their confidence in measurement skills. Those preferring AFROM endorsed it as quick, easy, and useful for informing treatment decisions. None of the participants rated PFROM as reliable or valid. See Figure 2 for a detailed account of the frequency of rationale for choosing a particular method.

In response to ‘*Has your preference changed over time?’*, many participants (*n* = 156, 77.2%) reported no change in preference for a method of measuring finger ROM over time, while 22.8% had changed their practice preferences. Justifications for practice changes were mapped onto components of the COM-B Model for Behaviour Change^22^ as described in Table 2.

**Table 2.** Mapping themes of participants change in preferences on the COM-B model.

COM-B	Themes	Illustrative Quotes
Participants who reported change in preferences		
Psychological capability	Preferences changed over time with more experience (knowledge/skills)	“I’ve made changes as my experience and learning evolves” – OT with 35 years of experience as a hand therapist
	Tailoring to individual (knowledge/skills)	“Use more than just one method depending on diagnosis and what I might expect to see.” – OT with 27 years of experience as a hand therapist
	Streamlining data entry (increased cognitive and practical skills)	“I used to always measure all joints. The computer data entry has made this tedious and difficult to analyse.” – OT with 37 years of experience as a hand therapist
	Prioritizing interventions over measurement (knowledge/skills)	“I have learned to spend my time treating the patient/educating the patient, and I selectively measure each joint when needed.” – OT with 9 years of experience as a hand therapist
Physical opportunity	More options and choices available (increased environmental resources)	“Increased flexibility with measuring choices and options seems to have expanded with experience and practice.” – OT with 10 years of experience as a hand therapist
	Time constraints (reduced environmental resources)	“Need to do what’s quick” – OT with 4 years of experience as a hand therapist
	Setting requirements (environmental resources)	“[I] used to work in an inpatient setting and more gross measurements were more appropriate in that environment” – PT with 14 years of experience as a hand therapist
Social opportunity	Remote care (shift in cultural norm and social cues)	“Remote care - yes!” – OT with 20 years of experience as a hand therapist
	Legal purposes and physician orders (requirement for professional practice)	“For legal purposes or if a ‘bigger picture view’ needs to be obtained by the doctor etc.” – OT with 17 years of experience as a hand therapist
	Patient health literacy (opportunity afforded by interpersonal influences and social cues)	“I never used to do TAM until a few years ago. It can be a great, simple number for patients to understand. *“My goal for you is about 250 degrees altogether. Last time you were only at about 95 degrees but today you are at 170 degrees. That’s so much better! Great job!”*” – OT with 31 years of experience as a hand therapist
Reflective motivation	Participation vs impairment focus (change in belief system)	“[I] used to be more obsessed with documenting numbers with goniometer now I’m more focused on functional return” – OT with 13 years of experience as a hand therapist
Participants who reported no change in preferences		
Psychological capability	Knowledge of clinimetric properties (knowledge/skills)	“I feel that specific measurements with goniometer are more reliable, especially if a different therapist will see patient.” – OT with 14 years of experience as a hand therapist
	To differentiate joint vs soft tissue limitation (knowledge/skills)	“I still prefer to take individual joint ROM measurements to indicate whether or not it’s joint stiffness, ligamentous, or other soft tissue issues” – OT with 28 years of experience as a hand therapist
	Inform treatment decision (knowledge/skills)	“[Goniometry] is the most informative, reliable, valid and therefore more useful for determining treatment interventions” – PT with 21 years of experience as a hand therapist
Physical capability	Practice history	“I always preferred gonio [metric] measurements” – OT with 5 years of experience as a hand therapist
Social opportunity	Easy for patient to understand (opportunity afforded by interpersonal influences and social cues)	“I measure ROM almost every visit and I feel the [patient] also enjoys knowing that their injured PIP joint that had only 35 degrees of flexion on day 1 is now 45° 2 days later and then 55°1 week later, etc. It gives me good objective measurements to justify my intervention and it involves patient more with therapy.” – OT with 24 years of experience as a hand therapist
	Lack of experience with multiple methods (lack of opportunity for knowledge acquisition)	“I’ve only been in hand [therapy] for 10 months” – OT with 10 months of experience as a hand therapist

**Figure 3.**
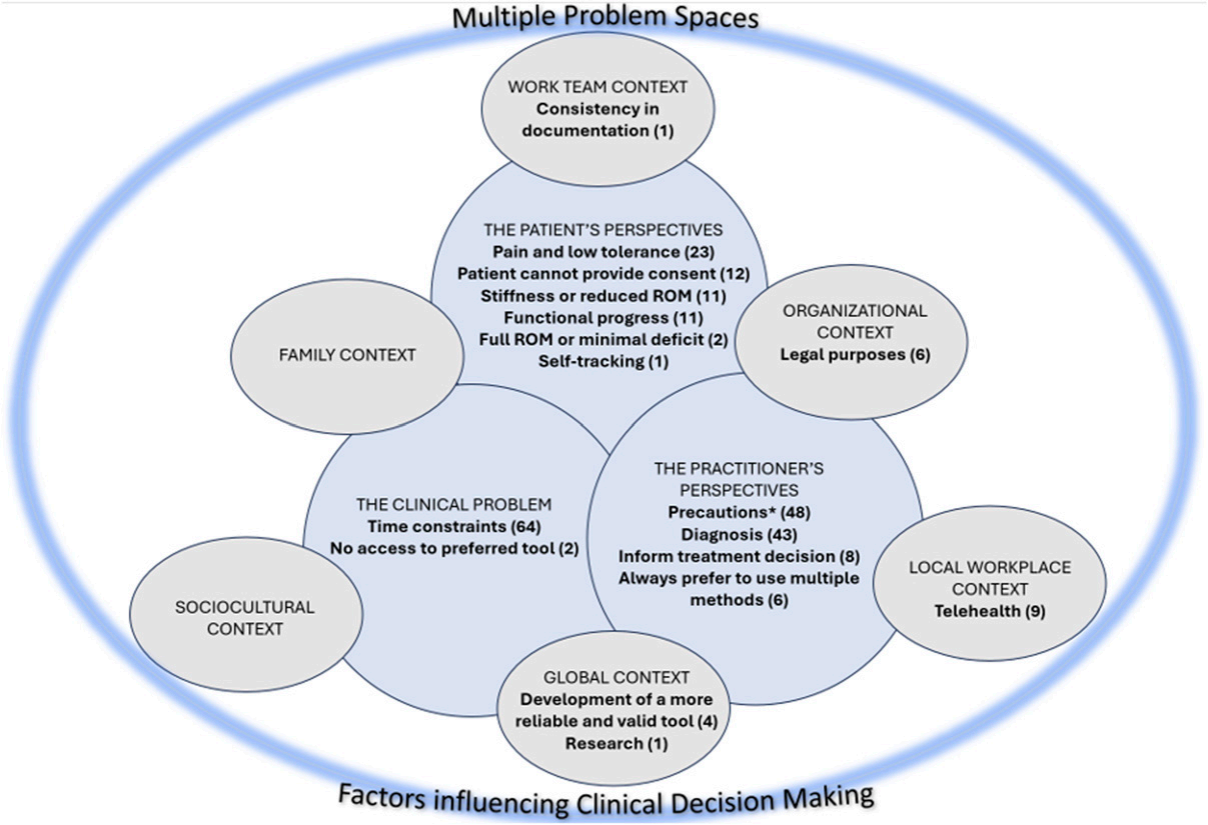
Factors influencing the decision to choose a different method of measuring ROM mapped on clinical decision making in multiple problem spaces *Pins, needles, k-wire, bulky dressing, edema, dorsal application of goniometer, open wounds, acute stage, infections, deformity, splint or cast.

In response to the question *When would you choose to use a different method than your preferred method?,* most participants reported they might use a different method based on clinical circumstances (*n* = 249, 97%) while very few (*n* = 6, 3%) reported they would never use a different method of measuring finger ROM. Data describing these clinical circumstances for using a different method were mapped on the CDM in Multiple Problem Spaces model^7^: see Figure 3.

A majority of participants reported that hand ROM measurements were never delegated to therapist assistants (*n* = 107, 57.2%) or there were no assistants available in their settings (*n* = 55, 29.4%). However, a small percentage delegated ROM measurements regularly (*n* = 9, 4.8%), occasionally (*n* = 8, 4.2%), or based on the skill set of the assistant (*n* = 8, 4.2%).

Two distinct techniques were reported for ruler placement when measuring FROM. Most participants (*n* = 149, 80%) instruct the patient to flex as far as possible, then place the ruler in the distal palmar crease at an angle to the fingertip. A smaller proportion of participants (*n* = 38, 20%) reported first placing the ruler at a 90-degree angle to the palm in the distal palmar crease, then instructing the patient to flex down the ruler as far as possible.

For client self-monitoring of finger flexion, participants most reported using FROM (*n* = 91, 48.7%); some participants adapted this technique by asking clients to mark on a card instead of using a ruler (*n* = 29, 15.5%). Additional self-monitoring techniques reported (*n* = 37, 19.7%) included taking photos, tracing, utilising objects (e.g. dowels, cylinders) for reference, comparing to the uninvolved hand, and printing out a large goniometer.

### Clinical reasoning used by hand therapists when measuring finger ROM

There was a total of 308 participants’ responses to the vignettes, where at least one of three vignettes were answered, however one response was excluded as the participant noted that they did not see such acute cases in their practice setting. Therefore *N* = 307 responses were analysed. Analysis of responses to the three sequential vignette segments suggested distinct patterns of clinical reasoning approaches (Table 3). For vignette part 1 [acute flexor tendon case], a majority (91.2%) of the participants followed procedural reasoning, selecting the measurement technique based on the client’s injury and healing. The remainder (8.8%) followed pragmatic reasoning based on organizational demands such as the surgeon’s protocol/orders and time constraints in the clinic. For part 2 of the vignette, most participants followed either pragmatic reasoning (48.8%) due to time constraints (preferring the use of FROM measures or only measuring the affected digit) or procedural reasoning (45.1%) based on the client’s injury and healing status. Few participants (5.8%) explicitly considered the client’s occupational demands through conditional reasoning, and only one participant focused on the client as a person by employing interactive reasoning. The majority of the responses (94.3%) to part 3 of the vignette followed procedural reasoning based on the client’s condition and limitations. A small subset (4.6%) of the responses employed pragmatic reasoning based on the surgeon’s requirements. Two responses used conditional reasoning to consider the client’s occupational demands: one response followed interactive reasoning, considering the client’s goals.

**Table 3.** Frequency and percentages of clinical reasoning to individual vignettes.

Type of reasoning used	In Vignette part 1	In Vignette part 2	In Vignette part 3
	Frequency (%)	Frequency (%)	Frequency (%)
Procedural	256 (91.1)	127 (32.3)	265 (94.3)
Pragmatic	25 (8.9)	136 (48.4)	13 (4.6)
Conditional	0	17 (6.0)	2 (0.7)
Interactive	0	1	1 (0.4)

For the next step of analysis, 281 complete responses were included in the overall analysis of the multi-stage vignette. Since many participants employed more than one type of clinical reasoning in their responses to the multi-stage vignettes, a total of 13 categories were found to describe these combinations (see table in supplemental material). 42.4% of participants followed a completely procedural approach focusing on the injury, stage of healing, and client limitations across the 3 visits described in the vignette. Many participants (38.4%) belonged to the Procedural/Pragmatic/Procedural category, changing their measurement technique due to the time constraint presented in the second scenario. Less than five percent (4.6%) of the responses fell into the category Procedural/Conditional/Procedural. This group of participants highlighted the importance of patient education and a home exercise program based on the client’s occupational demands.

## Discussion

This study explored the preferred finger ROM measurement techniques used by hand therapists, factors influencing their clinical decision-making, and reasoning processes employed when faced with practice-based measurement scenarios. The results showed clinicians frequently use multiple methods mainly goniometry of individual joints, AFROM, and TAM/TPM, and that different contextual factors contribute to these practice patterns. Additionally, clinicians appear to employ distinct patterns of clinical reasoning, mainly procedural and pragmatic reasoning, relative to finger ROM measurement scenarios. Together, these findings highlight variations in both the practice patterns and practice-based clinical reasoning.

The ASHT’s Clinical Assessment Recommendations^4^ present a comprehensive range of tools and techniques for measuring finger motion. While their synthesis illustrates many options with estimates of psychometric properties, information is lacking regarding the specific methods utilised in clinical practice. Conversely, the British Association of Hand Therapists (BAHT) recommends measuring TAM after flexor tendon injury.^26^ Our study contributes to understanding the current state of clinical practice through self-reported practice concerning the measurement of finger ROM.

A 2019 study ^27^ survey of certified hand therapists provided a broad analysis of the tools and techniques used in hand rehabilitation, classifying measuring active and passive ROM as an entry-level critical technique performed frequently (at least monthly to weekly). In contrast, we focussed specifically on finger ROM measurement methods and the influence of practice context and clinical reasoning on this assessment area. Unsurprisingly, measuring individual finger joints with a goniometer was the most preferred and frequently used measurement method for assessing finger ROM among hand therapists, aligning with previous work .^28–30^ However, while performing joint ROM tests falls within the scope of practice guidelines for occupational therapy assistants (OTA) and physiotherapy assistants (PTA) practice ,^31,32^ the majority of therapists in our study did not delegate finger ROM measurement tasks to therapy assistants. This is concordant with a survey study of PTs, where 25-69% of respondents did not think measurements were appropriate to delegate.^33^ However, two other survey studies of PTs reported performing joint ROM is a unique and complex task frequently delegated to PTAs^34^ or may be considered low complexity and thus delegated frequently to PTAs with varying amounts of supervision.^35^ Notably, these studies are older physical therapy literature focused on perceptions of the PTA role: none were specific to hand therapy or occupational therapy.

AFROM was used frequently to measure finger ROM, and endorsed as quick, easy, and useful in informing treatment decisions. This rationale was congruent with changing preferences due to the importance of tracking functional progress, time constraints, and spending more time on treatment. Further, we noted participants asked clients to self-monitor progress using the AFROM method or a variation thereof. Many participants also selected the AFROM measure in response to time pressures in the vignette. In contrast, PFROM was largely reported as rarely or never used; less than 1% preferred to use PFROM. None of the participants rated PFROM as reliable or valid. This can be attributed to limited literature^4^ on the psychometric properties of the FROM.

Our analysis of individual vignette segments suggested therapists focused on different aspects of the scenario presented to them and proceeded with different types of clinical reasoning. In our collective analysis of all 3 vignettes, we saw patterns similar to Mattingly and Fleming’s description of the three tracks of reasoning, which describes the use of more than one type of reasoning simultaneously where “…therapists could process or analyse different aspects of the problem almost simultaneously, using different styles of thinking…”.^36^ This flexibility was evident in the 13 different combinations of reasoning. Indeed, it may be that with actual clients, therapists would tacitly engage in more conditional and/or interactive reasoning; but they did not explicate this in their vignette responses. The three-part vignettes provided a scenario with specific questions, which may have constrained responses. In contrast, in the survey section addressing general measurement choices, participants were asked open-ended descriptive questions.

### Limitations and recommendations for future research

The timing of the survey distribution during the early stages of the COVID-19 pandemic holds importance. It coincided with substantial changes in healthcare and research, which had a noticeable impact on the survey roll-out and findings. First, questions were not made mandatory as we wanted to be flexible, anticipating that people were experiencing stressful work instability and disrupted routines. Consequently, the total number of responses for each question varied. While we reached our target response rate of 12.5% for total responses, complete responses fell below this standard. Low response rates may have led to limited use of certain question response options, which may further reflect the lack of in-depth cognitive debriefing for practice-related questions. Secondly, with the increased reliance on remote assessments and telehealth platforms, the utilisation of smartphone apps for measuring finger ROM may have gained popularity over the course of the pandemic.^37–40^ This contrasts with our results generated during the early months of 2020 where no participants reported using a smartphone app to measure finger ROM. Future practice surveys may examine if there is an increased uptake of measurement apps in hand therapy. Our findings also illuminate opportunities for future clinical measurement research: there is a need for more evidence on the psychometric properties of AFROM given the frequency of use in practice.

One of the limitations of survey research (compared to other forms of qualitative data collection) is the inability to probe for more insight into interesting or unusual findings.^8^ One such illustration in our work is the finding that while the majority of therapists had an assistant in their setting, they rarely or never delegated measurement of finger ROM to the assistant. Future research could explore the barriers to utilisation of assistants in the full scope of practice in a hand therapy setting and consider if this is unique to North American practice.

A further limitation of the study is that the majority of the respondent cohort represented the North American socioeconomic group (i.e. Canada and USA): the preferences for measuring ROM may not be fully representative of hand therapists across the globe, where clinical reasoning may be influenced by different settings e.g. culture and resource.

Another limitation of our survey is, that although we referred to finger and standard goniometers, we did not define them explicitly in the survey. Further, our fixed-choice questions around finger ROM measurement practices included multiple measurement strategies. However, since some of the choices were summary scores of individual joint measures, the response options were therefore not mutually exclusive and may have been interpreted differently. In both of these cases, we assumed participants would understand the difference between these measurement methods/strategies.

Clinical reasoning is a multifaceted process involving intricate cognitive dynamics.^7^ However, the vignettes employed in this study were not explicitly developed with any theory or framework in mind: instead, theory was applied at the analysis stage. Consequently, the vignettes may have failed to comprehensively capture the complexity of clinical reasoning, potentially limiting the depth of insights gained. Moreover, vignettes may not fully encompass the real-world complexity and ambiguity encountered by clinicians. The findings on clinical reasoning derived from our vignette analysis may be constrained by the deductive approach applied.^21^ Another limitation of the study is that the vignette scenarios focused on acute, post-operative flexor tendon injury measurement and rehabilitation. However, our findings suggest tendon injuries are seen occasionally rather than routinely, so the vignette may not have been well targeted to elicit reflections on daily practice.

## Conclusion

This study showed clinicians frequently use multiple methods to measure finger ROM in clinical practice, and that different contextual factors contribute to these practice patterns. Additionally, clinicians appear to employ distinct patterns of clinical reasoning relative to finger ROM measurement scenarios.

This study provides insight into the current professional practice patterns and elucidates variations for consideration by therapist educators and clinical preceptors. The findings also identify opportunities for improving evidence for clinical measurement methods and evidence implementation relative to existing knowledge.

## Supplemental Material


Supplemental Material - How is range of motion of the fingers measured in hand therapy practice? A survey study
Supplemental Material for How is range of motion of the fingers measured in hand therapy practice? A survey study by Zeal Kadakia, Sandra VanderKaay, Ayse Kuspinar and Tara Packham in Hand Therapy.
