# Supplementary material for: How is range of motion of the fingers measured in hand therapy practice? A survey study
Source: Hand Ther. 2024 Apr 22;29(3):112–23. doi: 10.1177/17589983241237780 (PMC11378534; doi:10.1177/17589983241237780)
Supplement: Supplemental Material - How is range of motion of the fingers measured in hand therapy practice? A survey study [file sj-pdf-1-hth-10.1177_17589983241237780.pdf]

Supplementary material

Table: Frequency and percentages of clinical reasoning categories. (N=281)

**Clinical reasoning category**	**Frequency (%)**
Procedural/Procedural/Procedural	119 (42.35%)
Procedural/Pragmatic/Procedural	108 (38.43%)
Pragmatic/Pragmatic/Procedural	18 (6.41%)
Procedural/Conditional/Procedural	13 (4.63%)
Procedural/Pragmatic/Pragmatic	7 (2.49%)
Pragmatic/Procedural/Procedural	5 (1.78%)
Procedural/Conditional/Pragmatic	3 (1.07%)
Procedural/Procedural/Pragmatic	2 (0.71%)
Procedural/Pragmatic/Conditional	2 (0.71%)
Procedural/Procedural/Interactive	1 (0.36%)
Procedural/Interactive/Procedural	1 (0.36%)
Pragmatic/Pragmatic/Pragmatic	1 (0.36%)
Pragmatic/Conditional/Procedural	1 (0.36%)

Figure A: Visual Representation of Codes Within the Four-Part Framework of Clinical Reasoning

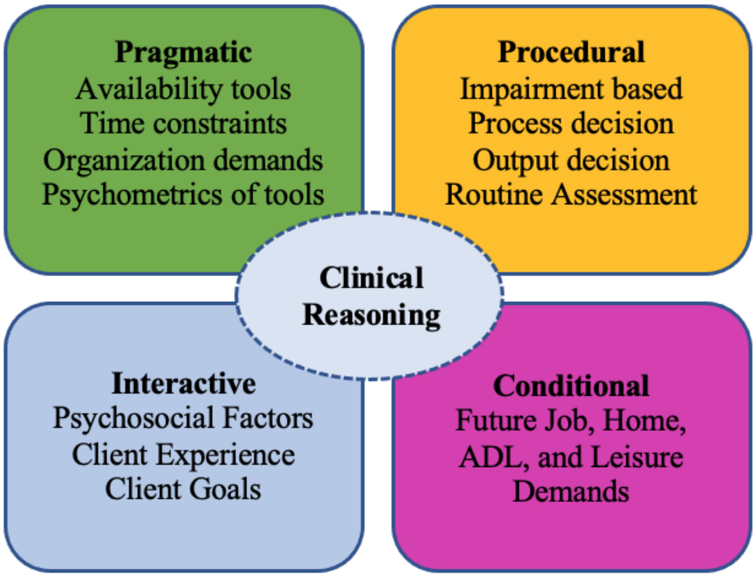

Figure B: Flow chart of participation

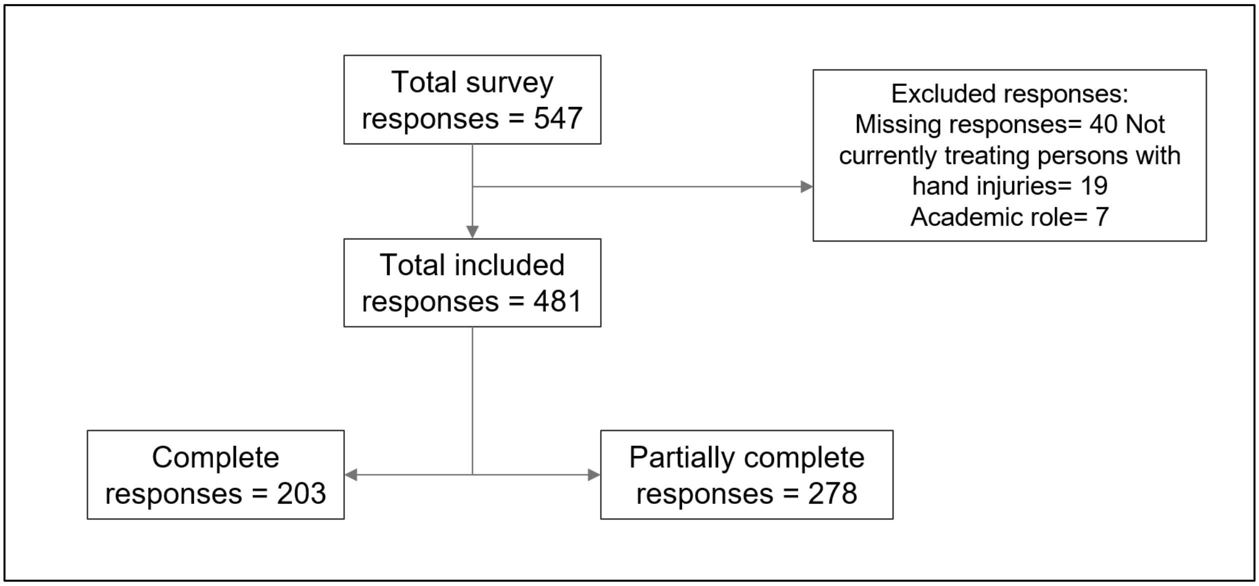

Graph A: Frequency of upper extremity diagnoses primarily seen across practice settings.

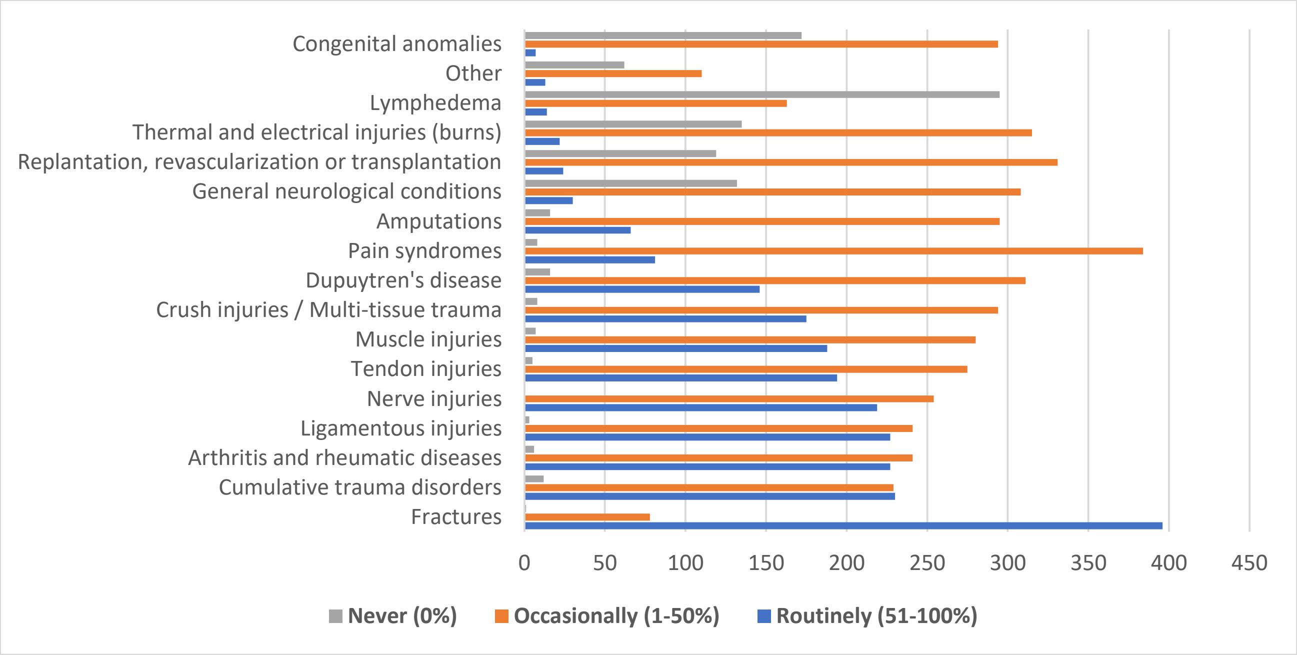

Graph B: Frequency of preference of any one finger ROM measurement method (N=216)

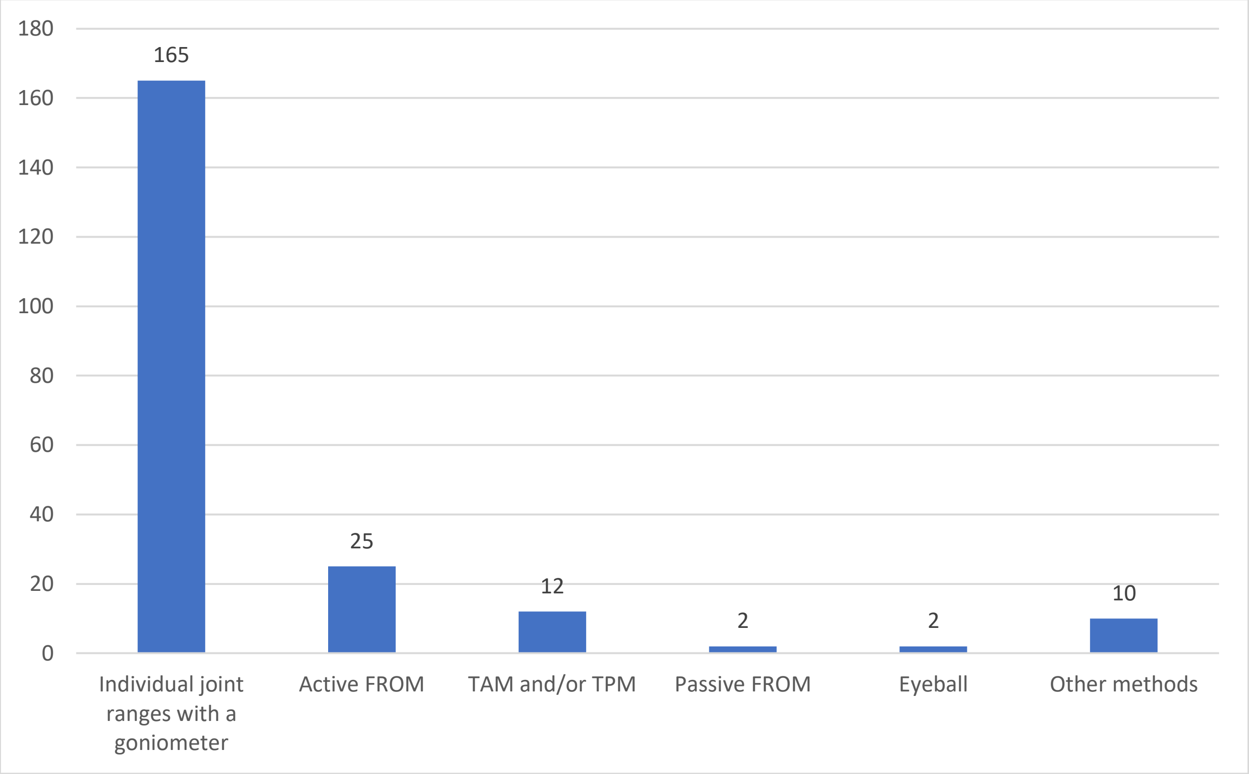

Appendix A

CHERRIES and CROSS checklists for reporting survey studies

**Checklist for Reporting Results of Internet E-Surveys (CHERRIES)**			
**Item Category**	**Checklist Item**	**Explanation**	**Reported on page**
Design	Describe survey design	Describe target population, sample frame. Is the sample a convenience sample? (In "open" surveys this is most likely.)	3-4
IRB approval and informed consent process	IRB approval	Mention whether the study has been approved by an IRB.	4
	Informed consent	Describe the informed consent process. Where were the participants told the length of time of the survey, which data were stored and where and for how long, who the investigator was, and the purpose of the study?	4
	Data protection	If any personal information was collected or stored, describe what mechanisms were used to protect unauthorized access.	4
Development and pre-testing	Development and testing	State how the survey was developed, including whether the usability and technical functionality of the electronic questionnaire had been tested before fielding the questionnaire.	3
Recruitment process and description of the sample having access to the questionnaire	Open survey vs closed survey	An "open survey" is a survey open for each visitor of a site, while a closed survey is only open to a sample which the investigator knows (password-protected survey).	4
	Contact mode	Indicate whether or not the initial contact with the potential participants was made on the Internet. (Investigators may also send out questionnaires by mail and allow for Web-based data entry.)	4
	Advertising the survey	How/where was the survey	4

		announced or advertised? Some examples are offline media (newspapers), or online (mailing lists – If yes, which ones?) or banner ads (Where were these banner ads posted and what did they look like?). It is important to know the wording of the announcement as it will heavily influence who chooses to participate. Ideally the survey announcement should be published as an appendix.	
Survey administration	Web/Email	State the type of e-survey (eg, one posted on a Web site, or one sent out through e-mail). If it is an e-mail survey, were the responses entered manually into a database, or was there an automatic method for capturing responses?	4
	Context	Describe the Web site (for mailing list/newsgroup) in which the survey was posted. What is the Web site about, who is visiting it, what are visitors normally looking for? Discuss to what degree the content of the Web site could pre-select the sample or influence the results. For example, a survey about vaccination on a anti-immunization Web site will have different results from a Web survey conducted on a government Web site	4
	Mandatory/Voluntary	Was it a mandatory survey to be filled in by every visitor who wanted to enter the Web site, or was it a voluntary survey?	4
	Incentives	Were any incentives offered (eg, monetary, prizes, or non-monetary incentives such as an offer to provide the survey results)?	No incentives offered
	Time/date	In what timeframe were the data collected?	4
	Randomization	To prevent biases items can be randomized or alternated.	N/A
	Adaptive questioning	Use adaptive questioning (certain items, or only conditionally displayed based on responses to other items) to reduce number and complexity of the	N/A

		questions.	
	Number of Items	What was the number of questionnaire items per page? The number of items is an important factor for the completion rate.	3
	Number of screens (pages)	Over how many pages was the questionnaire distributed? The number of items is an important factor for the completion rate.	3
	Completeness check	It is technically possible to do consistency or completeness checks before the questionnaire is submitted. Was this done, and if "yes", how (usually JAVAScript)? An alternative is to check for completeness after the questionnaire has been submitted (and highlight mandatory items). If this has been done, it should be reported. All items should provide a non-response option such as "not applicable" or "rather not say", and selection of one response option should be enforced.	N/A
	Review step	State whether respondents were able to review and change their answers (eg, through a Back button or a Review step which displays a summary of the responses and asks the respondents if they are correct).	N/A
Response rates	Unique site visitor	If you provide view rates or participation rates, you need to define how you determined a unique visitor. There are different techniques available, based on IP addresses or cookies or both.	4
	View rate (Ratio of unique survey visitors/unique site visitors)	Requires counting ratio of unique survey visitors divided by unique site visitors. It is not unusual to have view rates of less than 0.1 % if the survey is voluntary.	N/A
	Participation rate (Ratio of unique visitors who agreed to participate/unique first survey page visitors)	Count the unique ratio of those who agreed to participate divided by unique first survey page visitors. This can also be called "recruitment" rate.	N/A
	Completion rate (Ratio of users who	The ratio of the number of people who finished the survey divided by	N/A

	finished the survey/users who agreed to participate)	those who agreed to participate. This is a measure for attrition. Note that “completion” can involve leaving questionnaire items blank. This is not a measure for how completely questionnaires were filled in. (If you need a measure for this, use the word “completeness rate”.)	
Preventing multiple entries from the same individual	Cookies	Indicate whether cookies were used to assign a unique user identifier to each client computer. If so, mention the page on which the cookie was set and read, and how long the cookie was valid. Were duplicate entries avoided by preventing users access to the survey twice; or were duplicate database entries having the same user ID eliminated before analysis? In the latter case, which entries were kept for analysis (eg, the first entry or the most recent)?	N/A
	IP check	Indicate whether the IP address of the client computer was used to identify potential duplicate entries from the same user. If so, mention the period of time for which no two entries from the same IP address were allowed (eg, 24 hours). Were duplicate entries avoided by preventing users with the same IP address access to the survey twice; or were duplicate database entries having the same IP address within a given period of time eliminated before analysis? If the latter, which entries were kept for analysis (eg, the first entry or the most recent)?	4
	Log file analysis	Indicate whether other techniques to analyze the log file for identification of multiple entries were used. If so, please describe.	N/A
	Registration	In “closed” (non-open) surveys, users need to login first and it is easier to prevent duplicate entries from the same user. Describe how this was done. For example, was the survey	N/A

		never displayed a second time once the user had filled it in, or was the username stored together with the survey results and later eliminated? If the latter, which entries were kept for analysis (eg, the first entry or the most recent)?	
Analysis	Handling of incomplete questionnaires	Were only completed questionnaires analyzed? Were questionnaires which terminated early (where, for example, users did not go through all questionnaire pages) also analyzed?	5
	Questionnaires submitted with an atypical timestamp	Some investigators may measure the time people needed to fill in a questionnaire and exclude questionnaires that were submitted too soon. Specify the timeframe that was used as a cut-off point, and describe how this point was determined.	N/A
	Statistical correction	Indicate whether any methods such as weighting of items or propensity scores have been used to adjust for the non-representative sample; if so, please describe the methods.	N/A

**Checklist for Reporting Of Survey Studies (CROSS)**		
**Item/section**	**Item description**	**Reported on page**
Title and abstract	1a<br>State the word "survey" along with a commonly used term in title or abstract to introduce the study's design.	1
	1b<br>Provide an informative summary in the abstract, covering background, objectives, methods, findings/results, interpretation/discussion, and conclusions.	1
Introduction		
Background	2<br>Provide a background about the rationale of study, what has been previously done, and why this survey is needed.	2
Aim	3<br>Identify specific purposes, aims, goals, or objectives of the study.	2-3
Methods		
Study design	4<br>Specify the study design in the methods section with a	3

	commonly used term (e.g., cross-sectional or longitudinal).	
Data collection methods	5a<br>Describe the questionnaire (e.g., number of sections, number of questions, number and names of instruments used).	3
	5b<br>Describe all questionnaire instruments that were used in the survey to measure particular concepts. Report target population reported validity and reliability information, scoring/classification procedure, and reference links (if any).	3-4
	5c<br>Provide information on pretesting of the questionnaire, if performed (in the article or in an online supplement). Report the method of pretesting, number of times questionnaire was pre-tested, number and demographics of participants used for pretesting, and the level of similarity of demographics between pre-testing participants and sample population.	3
	5d<br>Questionnaire, if possible, should be fully provided (in the article, or as appendices or as an online supplement).	Appendix B
Sample characteristics	6a<br>Describe the study population (i.e., background, locations, eligibility criteria for participant inclusion in survey, exclusion criteria).	4
	6b<br>Describe the sampling techniques used (e.g., single stage or multistage sampling, simple random sampling, stratified sampling, cluster sampling, convenience sampling). Specify the locations of sample participants whenever clustered sampling was applied.	4
	6c<br>Provide information on sample size, along with details of sample size calculation.	4
	6d<br>Describe how representative the sample is of the study population (or target population if possible), particularly for population-based surveys.	4
Survey administration	7a<br>Provide information on modes of questionnaire administration, including the type and number of contacts, the location where the survey was conducted (e.g., outpatient room or by use of online tools, such as SurveyMonkey).	4
	7b<br>Provide information of survey's time frame, such as periods	4

	of recruitment, exposure, and follow-up days.	
	7c<br>Provide information on the entry process:<br>–>For non-web-based surveys, provide approaches to minimize human error in data entry.<br>–>For web-based surveys, provide approaches to prevent "multiple participation" of participants	4
Study preparation	8<br>Describe any preparation process before conducting the survey (e.g., interviewers' training process, advertising the survey).	3
Ethical considerations	9a<br>Provide information on ethical approval for the survey if obtained, including informed consent, institutional review board [IRB] approval, Helsinki declaration, and good clinical practice [GCP] declaration (as appropriate).	4
	9b<br>Provide information about survey anonymity and confidentiality and describe what mechanisms were used to protect unauthorized access.	4
Statistical analysis	10a<br>Describe statistical methods and analytical approach. Report the statistical software that was used for data analysis.	4-5
	10b<br>Report any modification of variables used in the analysis, along with reference (if available).	N/A
	10c<br>Report details about how missing data was handled. Include rate of missing items, missing data mechanism (i.e., missing completely at random [MCAR], missing at random [MAR] or missing not at random [MNAR]) and methods used to deal with missing data (e.g., multiple imputation).	8
	10d<br>State how non-response error was addressed.	N/A
	10e<br>For longitudinal surveys, state how loss to follow-up was addressed.	N/A
	10f<br>Indicate whether any methods such as weighting of items or propensity scores have been used to adjust for non-representativeness of the sample.	N/A
	10g<br>Describe any sensitivity analysis conducted.	N/A
Results		
Respondent characteristics	11a<br>Report numbers of individuals at each stage of the study.	6

	Consider using a flow diagram, if possible.	
	11b<br>Provide reasons for non-participation at each stage, if possible.	N/A
	11c<br>Report response rate, present the definition of response rate or the formula used to calculate response rate.	N/A
	11d<br>Provide information to define how unique visitors are determined. Report number of unique visitors along with relevant proportions (e.g., view proportion, participation proportion, completion proportion).	4
Descriptive results	12<br>Provide characteristics of study participants, as well as information on potential confounders and assessed outcomes.	6
Main findings	13a<br>Give unadjusted estimates and, if applicable, confounder-adjusted estimates along with 95% confidence intervals and p-values.	N/A
	13b<br>For multivariable analysis, provide information on the model building process, model fit statistics, and model assumptions (as appropriate).	N/A
	13c<br>Provide details about any sensitivity analysis performed. If there are considerable amount of missing data, report sensitivity analyses comparing the results of complete cases with that of the imputed dataset (if possible).	N/A
Discussion		
Limitations	14<br>Discuss the limitations of the study, considering sources of potential biases and imprecisions, such as non-representativeness of sample, study design, important uncontrolled confounders.	10
Interpretations	15<br>Give a cautious overall interpretation of results, based on potential biases and imprecisions and suggest areas for future research.	10-11
Generalizability	16<br>Discuss the external validity of the results.	N/A
Other sections		
Funding source	17<br>State whether any funding organization has had any roles in the survey's design, implementation, and analysis.	N/A

Conflict of interest	18<br>Declare any potential conflict of interest.	None
Acknowledgements	19<br>Provide names of organizations/persons that are acknowledged along with their contribution to the research.	

Appendix B : Full Survey
*Questions NOT reported or analysed in this survey are in red ink.

Hello hand therapy colleagues; This survey was initially developed many months ago, before any of us were aware of the emerging challenges from COVID19. We understand many of you will have experienced and will continue to experience dramatic changes in your practice as a result of the current pandemic. However, we decided to launch the survey as planned because 1) it is a student research project intended to inform ongoing coursework, and 2) it is not directly impacted by physical distancing restrictions (as is the case for many other clinical research endeavors at this time). We are therefore inviting you to complete the following practice survey using the time frame of January 2020 as the reference point for your answers. Please tell us about your practice relative to where you were working in January, and think about the clinical cases relative to how you were seeing your caseload in that time frame. Thank you for your support of this research project.

Best wishes for health and resilience,

How is range of motion measured in hand therapy practice? A survey study.

Part One: Demographics

1. What is your current age? (*dropdown box)*

2. With what gender do you most closely identify?

○ Male

○ Female

○ Non-binary

○ Fluid

○ Prefer to self-identify as ____________________

○ Prefer not to answer

3. What is your profession? (*Select all that apply.*)

☐ Occupational therapist

☐ Physical therapist or Physiotherapist

☐ Kinesiologist

- [ ] Athletic therapist
- [ ] Orthopedic surgeon
- [ ] Plastic surgeon
- [ ] Physician
- [ ] Registered nurse
- [ ] Occupational therapist assistant
- [ ] Physical therapist assistant
- [ ] Other ______________________

4. What was your entry-level professional degree?

- ○ Assistant diploma / certificate
- ○ Associate's degree
- ○ Bachelor's degree
- ○ Master's degree
- ○ Doctoral degree
- ○ N/A: I am an entry-level student

5. What is your highest level of education?

- ○ Associate's degree
- ○ Bachelor's degree
- ○ Clinical master's degree (e.g. MPT, MOT, MScPT, MScOT)
- ○ Master's degree (academic: course or thesis based i.e. MSc, MEd)
- ○ Clinical doctoral degree (e.g. DPT, OTD)
- ○ Academic doctoral degree (e.g. PhD, EdD, ScD)

6. How many years have you been licensed/registered as a health professional?

[*dropdown box for whole numbers, also include not applicable*]

7. How many years have you identified as a hand therapist or practiced in a hand therapy role?

[*dropdown box for whole numbers, including less than one year and not applicable*]

8. Are you a certified hand therapist (CHT?)

- ○ Yes, current certification through the Hand Therapy Certification Commission
- ○ Yes, certified through another organization outside North America
- ○ No, but am pursuing certification
- ○ Not currently, but held previously
- ○ No
- ○ Not endorsed as a qualification in my jurisdiction

Display this question only if Question 7
Are you a certified hand therapist (CHT?) = Yes

What year did you become a CHT? ________________________________

9. Do you practice in the United States?

- ○ Yes
- ○ No

Display this question only if
Do you Practice in the Unites States = Yes

In which state(s) do you currently practice? (*Select all that apply. Use Ctrl to select multiple options in the list.*)
Alabama
Alaska etc
[other 50 states, D.C., and Puerto Rico]

Display this question only if
Do you Practice in the United States = No, and do you Practice in Canada = No

In what country or countries do you currently practice outside of the United States? ________________

10. Do you practice in Canada?

Display this question only if
Do you Practice in Canada = Yes

In which province(s) or territory do you currently practice? (*Select all that apply. Use Ctrl to select multiple options in the list.*)
Alberta
British Columbia etc

11. How would you classify your primary practice setting?

- ○ Urban
- ○ Suburban
- ○ Rural
- ○ Other (please specify) ______________________________

12. What is your current primary practice setting?

- ○ Hospital-based outpatient clinic
- ○ Academic-based hospital clinic setting (outpatient)
- ○ Physician-owned outpatient clinic
- ○ Therapist-owned outpatient clinic
- ○ Corporate-owned freestanding outpatient clinic
- ○ Private practice, independent contractor
- ○ Inpatient: Hospital
- ○ Inpatient: Rehabilitation or skilled nursing facility
- ○ Home health care system
- ○ Military Health System
- ○ Veterans Health Administration System
- ○ Academia
- ○ Research
- ○ Industry
- ○ Other _________________________________

Do you practice as a hand therapist in this setting?

O Yes O No

13. Of the hours you worked in the past month, what best describes the percentage of time spent on direct or indirect patient care (not teaching or administration) across all worked hours?

- ○ None
- ○ 1 - 25%
- ○ 26-50%
- ○ 51-75%
- ○ 76-99%
- ○ 100%

14. Approximately how many hours per week in the past month did you engage in direct clinical care with patients with upper extremity conditions across your practice settings?

- ○ 0-9 hours per week
- ○ 10-19 hours per week
- ○ 20-29 hours per week
- ○ 30-40 hours per week
- ○ More than 40 hours per week

15. In an average month, how often do you treat these age groups across your practice settings? (*Your answers do not need to add up to 100%)*

	Never (0%)	Rarely (1-25%)	Occasionally (26-50%)	Often (51-75%)	Routinely (76-100%)
Pediatrics (0-17)	○	○	○	○	○
Adults (18-64)	○	○	○	○	○
Seniors/Older Adults (65+)	○	○	○	○	○

16. In an average month, how often do you treat these conditions as the primary diagnosis across your practice settings?

	Never (0%)	Rarely (1-25%)	Occasionally (26-50%)	Often (51-75%)	Routinely (76-

					100%)
Amputations	○	○	○	○	○
Arthritis and rheumatic diseases	○	○	○	○	○
Congenital anomalies	○	○	○	○	○
Crush injuries / multi-tissue trauma	○	○	○	○	○
Cumulative trauma disorders	○	○	○	○	○
Dupuytren's disease	○	○	○	○	○
Fractures	○	○	○	○	○
General neurological conditions (e.g. stroke, Parkinson disease, multiple sclerosis, amyotrophic lateral sclerosis, spinal cord injuries, cerebral palsy, muscular dystrophy)	○	○	○	○	○
Ligamentous injury or instability	○	○	○	○	○
Lymphedema	○	○	○	○	○
Muscle strains or tears or avulsions (acute)	○	○	○	○	○
Nerve injuries (including compressions, palsies, repairs, grafts)	○	○	○	○	○
Pain syndromes (e.g. complex regional pain syndrome, fibromyalgia)	○	○	○	○	○
Replantation, revascularization or transplantation	○	○	○	○	○
Tendon injuries or surgeries (e.g. lacerations, transfers, ruptures)	○	○	○	○	○
Thermal and electrical injuries (burns)	○	○	○	○	○
Other ____________	○	○	○	○	○

The preceding questions were developed and pre-tested for accuracy and inclusivity. Do you have any comments or suggestions on how to improve demographic reporting?

Part Two: Practice Survey

Hand measurement practice scenarios (vignettes)

To understand how people think about measuring motion, we have 3 short scenarios (vignettes) we would like you to consider. At the end of each vignette, you will be asked several questions. You don't have to write in full sentences to answer, but try to include 2-3 points with the details you think are important.

VIGNETTE #1

This vignette has three parts. Please do not go back and change your answers to an earlier part based on the new information – we are interested in understanding how new information is used in clinical decision-making.

Part A

Mark is a 25 year-old graduate student who underwent 6 strand flexor tendon repairs 4 weeks ago to his left non-dominant hand. He lacerated both FDS and FDP to the index finger in zone 2 while cutting an avocado.   You last saw him 10 days ago when he came in for suture removal.

Please explain what finger measurements would you perform and why? Be specific (which joints, directions, digits, active vs. passive)

Part B

As you sit down with Mark, he tells you he is in a hurry as he is leaving later tonight to go present a poster of his research at a national conference.

How would this affect what measurements you would perform? Be specific on what you would modify (if anything) in this situation. Are there any factors you would consider to inform your plan?

Part C

You have been away on vacation for several weeks. On your first day back, Mark is the first client you see. He is now 6 weeks post repairs, and is going to see his hand surgeon as soon as he leaves your clinic.

What finger measurements would you perform and why? Be specific (which joints, directions, digits, active vs. passive).

VIGNETTE #2

Karim is a 45 year-old roofer who sustained partial thickness burns to the extensor surface of his right dominant hand and forearm one month ago.   He has been reluctant to look at or touch his arm and residual eschar is visible when you remove his bandages. His doctor has asked you to measure wrist movement.

What technique would you use to measure Karim's wrist? Be specific about goniometer placement, and anatomical reference points

What are the potential advantages and disadvantages of this choice?

VIGNETTE #3

Lyla is a 70 year-old woman who fell and broke her wrist 10 weeks ago, and was managed conservatively with 6 weeks of casting. She lives 2 hours away from the Hand Centre and has made good overall progress with a tailored home program. However, she is quite upset that her limited supination has made it difficult to play bridge with her friends as she has done every Thursday for the past 7 years.   Her son (a physiotherapist in another state) has suggested she should ask you about being fitted with a serial static orthosis for mobilization.

What technique would you use to measure Lyla's forearm rotation? Be specific about your choice of measurement instrument, instrument placement, and anatomical reference points

What are the potential advantages and disadvantages of this choice?

Part 3: Survey questions

When measuring motion in the hand and fingers, how often have you used the following measurement techniques in the past month?

- Individual, isolated joint ranges (MP, PIP, DIP) with a goniometer placed dorsally or volarly
- Individual, isolated joint ranges (MP, PIP, DIP) with a goniometer placed laterally
- Strickland or other summary score
- Total active motion (TAM) = a summary of active flexion AND extension
- Total passive motion (TPM) = a summary of passive flexion AND extension
- Functional range of motion (FROM) / pulp-to-palm / composite finger flexion (CFF) = **active** combined flexion measured with ruler as distance between fingertip and distal palmar crease (in cm)
- FROM / CFF **passive** = combined passive flexion measured with ruler as distance between fingertip and distal palmar crease (in cm)
- wire bending (solder wire shaped to finger)
- paper strip method
- ‘Eyeball’ or visual estimate
- Other ________________________________

Response options:

- used always
- used frequently
- used occasionally
- used rarely
- never used in past month
- I have never used that ever
- I don’t know what that is

Which one method do you prefer to use?

- Individual, isolated joint ranges (MP, PIP, DIP) with a goniometer placed dorsally or volarly
- Individual, isolated joint ranges (MP, PIP, DIP) with a goniometer placed laterally
- Strickland or other summary score
- Total active motion (TAM) = a summary of active flexion AND extension
- Total passive motion (TPM) = a summary of passive flexion AND extension
- Functional range of motion (FROM) / pulp-to-palm / composite finger flexion (CFF) = **active** combined flexion measured with ruler as distance between fingertip and distal palmar crease (in cm)
- FROM / CFF **passive** = combined passive flexion measured with ruler as distance between fingertip and distal palmar crease (in cm)
- wire bending (solder wire shaped to finger)
- paper strip method

- ‘Eyeball’ or visual estimate
- Other ___________________________________

Why do you prefer this method? Select your top 5 answers [options with comment box; select all that apply]

- No preference
- Quick and easy
- Useful to inform treatment decisions
- The equipment is available in my setting
- Less impact of pins or dressings
- Reliable
- Valid
- Responsive
- Reflective of function
- Confident in my measurement skill
- Setting requires me to do it this way
- Referring physician prefers
- Insurance requires/requests
- Client prefers
- Past/current mentor prefers

When would you choose to use a different method than your preferred method?

Has your preference changed over time?

What equipment do you use for your preferred method?

- finger goniometer
- standard goniometer
- electronic goniometer
- smartphone app
- Other

When measuring movement of the thumb, how often do you use the following measurement techniques?

- individual joints (MP, IP) with goniometer
- TAM or TPM
- Kapandji index (0-10)
- Composite opposition **active** combined flexion/rotation measured with ruler as distance between tip of thumb and base of D5 (in cm)
- Pollexograph
- CMC abduction and extension with goniometer
- CMC abduction and extension measured as distance in cm with a ruler
- ‘Eyeball’ or visual estimate in degrees
- ‘Eyeball’ or visual estimate as a category (i.e. full, ½, etc.)

- Other___________________

Response options:

- used always
- used frequently
- used occasionally
- used rarely
- never used in past month
- I have never used that ever
- I don’t know what that is

Which one method do you prefer to use?

- individual joints (MP, IP) with goniometer
- TAM or TPM
- Kapandji index (0-10)
- Composite opposition **active** combined flexion/rotation measured with ruler as distance between tip of thumb and base of D5 (in cm)
- Pollexograph
- CMC abduction and extension with goniometer
- CMC abduction and extension measured as distance in cm with a ruler
- ‘Eyeball’ or visual estimate in degrees
- ‘Eyeball’ or visual estimate as a category (i.e. full, ½, etc.)
- Other___________________

Why do you prefer this method? Select your top 5 answers [options with comment box; select up to 5]

- No preference
- Quick and easy
- Useful to inform treatment decisions
- The equipment is available in my setting
- Less impact of pins or dressings
- Reliable
- Valid
- Responsive
- Reflective of function
- Confident in my measurement skill
- Setting requires me to do it this way
- Referring physician prefers
- Insurance requires/requests
- Client prefers
- Past/current mentor prefers

When would you choose to use a different method than your preferred method?

Has your preference changed over time?

What equipment do you use for your preferred method?

- finger goniometer
- standard goniometer
- electronic goniometer
- smartphone app
- Other

When measuring wrist flexion and extension, how often do you use the following measurement techniques?

- Measured along ulnar border in forearm (stationary arm) with 5th MC as reference in hand (moving arm)
- Measured along ulnar border in forearm (stationary arm) with 3rd MC as reference in hand (moving arm)
- Measures along radial border in forearm (stationary arm) with 3rd MC as reference in hand (moving arm)
- Dorsal / Volar along central forearm (stationary arm) with 3rd MC as reference in hand (moving arm) (LaStayo technique)
- 'Eyeball' visual estimate in degrees
- 'Eyeball' visual estimate reported in fractions (i.e. full, ½, 1/3 etc.)
- Other_____________________________

Response options:

- used always
- used frequently
- used occasionally
- used rarely
- never used in past month
- I have never used that ever
- I don't know what that is

Which one method do you prefer to use?

- Measured along ulnar border in forearm (stationary arm) with 5th MC as reference in hand (moving arm)
- Measured along ulnar border in forearm (stationary arm) with 3rd MC as reference in hand (moving arm)
- Measures along radial border in forearm (stationary arm) with 3rd MC as reference in hand (moving arm)
- Dorsal / Volar along central forearm (stationary arm) with 3rd MC as reference in hand (moving arm) (LaStayo technique)
- 'Eyeball' visual estimate in degrees

- ‘Eyeball’ visual estimate reported in fractions (i.e. full, ½, 1/3 etc.)
- Other______________________________

Why do you prefer this method? Select your top 5 answers [options with comment box; select up to 5]

- No preference
- Quick and easy
- Useful to inform treatment decisions
- The equipment is available in my setting
- Less impact of pins or dressings
- Reliable
- Valid
- Responsive
- Reflective of function
- Confident in my measurement skill
- Setting requires me to do it this way
- Referring physician prefers
- Insurance requires/requests
- Client prefers
- Past/current mentor prefers

When would you choose to use a different method than your preferred method?

Has your preference changed over time?

What equipment do you use for your preferred method?

- finger goniometer
- standard goniometer
- electronic goniometer
- smartphone app
- Other

When measuring forearm pronation or supination, how often do you use the following measurement techniques?

- Moving arm of goniometer aligned at distal 1/3 of forearm with stationary arm pointing upwards or downwards
- Stationary arm of goniometer aligned with humerus and moving arm aligned with pencil held in fist
- Stationary arm of goniometer aligned with humerus and moving arm aligned with 3rd proximal phalanx (with D3 flexed to 90 degrees at MCP)
- Inclinometer on a handle
- LaStayo technique using the fishing weight hung through the axis of a goniometer and attached to a handle

- ‘Eyeball’ visual estimate reported in degrees
- ‘Eyeball’ visual estimate reported in fractions (1/3, ½, etc)
- Other________________________________

Response options:

- used always
- used frequently
- used occasionally
- used rarely
- never used in past month
- I have never used that ever
- I don’t know what that is

Which one method do you prefer to use?

- Moving arm of goniometer aligned at distal 1/3 of forearm with stationary arm pointing upwards or downwards
- Stationary arm of goniometer aligned with humerus and moving arm aligned with pencil held in fist
- Stationary arm of goniometer aligned with humerus and moving arm aligned with 3rd proximal phalanx (with D3 flexed to 90 degrees at MCP)
- Inclinometer on a handle
- LaStayo technique using the fishing weight hung through the axis of a goniometer and attached to a handle
- ‘Eyeball’ visual estimate reported in degrees
- ‘Eyeball’ visual estimate reported in fractions (1/3, ½, etc)
- Other________________________________

When would you choose to use a different method than your preferred method?

Has your preference changed over time?

What equipment do you use for your preferred method?

- finger goniometer
- standard goniometer
- electronic goniometer
- smartphone app
- Modified goniometer with fishing weight
- Other

How often do you measure and document your patients’ ROM?

- At initial evaluation only
- Every visit
- Most visits
- Occasionally or as needed
- As needed for insurance reimbursement

- As requested by physician/as needed for physician follow-up visits
- As stipulated by policy/record-keeping
- As stipulated by progression of rehabilitation protocol
- Rarely
- Never

Are ROM measurements ever delegated to therapist assistants in your setting?

- Yes, regularly
- Yes, occasionally
- Yes, but based on skill set of assistant
- No
- N/A, no assistant in my setting

Do you measure hyperextension when it is not associated with pathology (i.e. normal flexibility)?

If you measure functional ROM / composite finger flexion / pulp-to-palm distance, how do you place the ruler?

- Ruler placed at 90 degrees to the palm in the distal palmar crease and the patient is instructed to flex to the ruler, moving as far down as possible
- Patient is instructed to flex as far as possible, and the ruler is subsequently placed in distal palmar crease and angled to fingertip
- I do not use this measurement technique

What measurement techniques do you instruct clients in for self-monitoring of ROM?

- pulp-to-palm / functional ROM / composite finger flexion
- marking on a card oriented as for a pulp-to-palm measurement
- smartphone apps
- Other ___________
- I do not instruct clients in self-monitoring of ROM

Is there any movement measure(s) you do not feel confident when performing?

What barriers (if any) do you perceive to assessing ROM of the hand, wrist and forearm in your current practice setting

Is there anything else you would like to tell us about how you use or perform measurements of hand, wrist or forearm motion?
